# Effect of Harvest Time and Packing Density on the Quality and Clostridium in Maize Silage

**DOI:** 10.3390/microorganisms13092096

**Published:** 2025-09-09

**Authors:** Fan Yang, Dongqing Fu, Lihe Su, Xue Yu, Jiaying Lv, Chunhui Ma

**Affiliations:** Grassland Science, School of Animal Technology, Shihezi University, Shihezi 832000, China; fanyang_shz_edu@126.com (F.Y.); fudongqing2024@163.com (D.F.); lihesu2025@163.com (L.S.); 19530282802@163.com (X.Y.); lvjiayingsc@163.com (J.L.)

**Keywords:** *Clostridium*, harvest stage, packing density, quality, whole-plant maize silage

## Abstract

This study investigated whole-plant maize at three harvest stages: one-third milk line (ML, 1/3 ML), two-thirds ML (2/3 ML), and the mature stage. Two packing densities were applied: 350 kg/m^3^ (low-density group) and 700 kg/m^3^ (high-density group). Results showed that starch content increased significantly as the maize matured. The 2/3 ML stage exhibited a 34.0% increase in starch content compared to the 1/3 ML stage (27.96 g/kg dry matter: DM vs. 20.87 g/kg DM, *p* < 0.01), while the mature stage showed a 13.4% increase compared to the 2/3 ML stage (31.70 g/kg DM vs. 27.96 g/kg DM, *p* < 0.01). After 60 days of ensiling, DM loss was significantly lower in the high-density group compared to the low-density group (3.37% vs. 9.39%, *p* < 0.05). From day 7 to day 60 of fermentation, the lactic acid content in the high-density group was consistently higher than in the low-density group by 14.29%, 10.00%, 8.33%, and 9.68%, respectively (*p* < 0.01). The relative abundance of *Clostridium* in both groups gradually increased during the first 30 days of fermentation, peaking on day 30 (0.05% in the high-density group vs. 0.12% in the low-density group, *p* < 0.05), and declined thereafter. On day 30, the abundance of *Ruminiclostridium* was significantly lower in the high-density group compared to the low-density group (0.12% vs. 0.40%, *p* < 0.05). *Clostridium* was negatively correlated with lactic acid bacteria (R^2^ = −0.58, *p* < 0.01). It also showed negative correlations with pH, lactic acid, and acetic acid (R^2^ = −0.25, −0.23, and −0.09, respectively; *p* > 0.05), but a positive correlation with ammoniacal nitrogen (R^2^ = 0.28, *p* > 0.05). In conclusion, the 2/3 ML stage is the optimal harvest time for whole-plant maize. Additionally, a higher packing density can suppress spoilage-associated *Clostridium* and enhance silage quality.

## 1. Introduction

Whole-plant maize silage is a vital feed resource, particularly for ruminants such as cattle and sheep [1]. Several factors influence silage quality, with harvest stage, packing density, and microbial community dynamics being especially critical [2]. Proper management of these factors can significantly enhance fermentation quality, promote the proliferation of beneficial microorganisms, and ultimately improve the nutritional value of the feed. The harvest stage directly affects the nutritional composition of maize silage [3]. As maize matures, key nutritional components, such as dry matter (DM), water-soluble carbohydrates (WSC), neutral detergent fiber (NDF), acid detergent fiber (ADF), and crude protein (CP), undergo considerable changes [4]. Therefore, selecting the appropriate harvest stage is essential for optimizing the silage’s nutritional content.

Packing density also plays a pivotal role in shaping microbial structure and influencing silage quality. A low packing density introduces more oxygen into the silage mass, encouraging the metabolism of aerobic microorganisms and impairing fermentation quality [5]. In contrast, a high packing density promotes anaerobic conditions, favoring the growth of lactic acid bacteria and enhancing fermentation quality [6]. Thus, selecting an appropriate packing density is essential for ensuring effective ensiling.

Microbial community dynamics are another key determinant of silage quality [7]. During ensiling, the activity of microorganisms such as lactic acid bacteria and *Clostridium* significantly influences the microbial ecosystem. When lactic acid bacteria dominate, their rapid production of lactic acid lowers the pH, thereby inhibiting harmful microbes and preserving silage quality [8]. Conversely, anaerobic microorganisms like *Clostridium* spp. can produce undesirable metabolites such as butyric acid and acetone, which deteriorate fermentation quality [9]. As fermentation progresses, microbial interactions shift, ultimately stabilizing into an anaerobic microbial community [10]. Packing density affects this succession and interplay of microbial populations, thereby influencing the overall fermentation outcome.

Therefore, this study aims to examine the effects of harvest stage and packing density on the fermentation quality of whole-plant maize silage, with a particular focus on the presence of *Clostridium*. By analyzing variations in nutritional composition, fermentation characteristics, and microbial community dynamics, this research provides a theoretical foundation for effective silage management and offers valuable insights for enhancing feed quality.

## 2. Materials and Methods

### 2.1. Experimental Materials

The maize used in this experiment was cultivated at the Forage Experiment Station of Shihezi University in Xinjiang, located at 44°21′4″ N latitude, 85°57′35″ E longitude, and an altitude of 420 m. The region experiences a continental temperate arid to semi-arid climate, with an average annual precipitation of 233 mm and approximately 2740.6 h of sunshine per year. Whole-plant maize was harvested at three developmental stages: one-third milk line (ML, 1/3 ML), two-thirds ML (2/3 ML), and mature stage. For each harvest stage, the maize plants were chopped into 1–3 cm lengths, and five replicates were prepared. Plastic silage tanks with a total volume of 25 kg/m^3^ were used for ensiling. For the main fermentation experiment, chopped whole-plant maize from the 2/3 ML stage was promptly transported to the laboratory and packed into plastic fermenters (Table 1) at two target packing densities: 350 kg/m^3^ and 700 kg/m^3^.

Samples were uniformly grouped using the quartering method to ensure consistency across all fermenters. In total, 30 silage samples were prepared (2 packing densities × 5 fermentation time points × 3 replicates). Silage tanks were opened on days 1, 7, 15, 30, and 60. The top and bottom 10 cm of each sample were discarded, and the material was collected from the middle section, thoroughly mixed, and divided into two portions: one for silage quality analysis and the other for microbial diversity analysis.

### 2.2. Experimental Methods

#### 2.2.1. Determination of Nutritional Composition

Nutritional composition was determined following the method described by Li et al. [11]. DM content was measured by drying fresh whole-plant maize and silage samples at 65 °C for 72 h. After grinding and sieving the samples through a 1 mm screen, CP content was analyzed using a Kjeldahl nitrogen analyzer (K9840, Shandong Haineng Scientific Instrument Co., Ltd., Jinan, China). NDF and ADF contents were measured using an ST116A fiber analyzer (Shandong Shengtai Instrument Co., Ltd., Jinan, China). WSC were determined using the anthrone reagent method [12], and starch content was measured by the dual-enzyme hydrolysis method [13].

#### 2.2.2. Determination of Fermentation Quality

To assess fermentation quality, 20 g of whole-plant maize silage was collected from the fermenters on days 1, 7, 15, 30, and 60. Each sample was mixed with 180 mL of deionized water and thoroughly shaken. The mixture was then stored at 4 °C for 24 h before being filtered through gauze. The pH of the resulting filtrate was measured immediately using a pH meter. A portion of the filtrate was used to determine the concentrations of lactic acid, acetic acid, propionic acid, and butyric acid using a high-performance liquid chromatograph (HPLC) (Agilent 1200, Shandong Jielun Technology Products Co., Ltd., Jinan, China) [14]. The filtrate was centrifuged at 12,000 rpm for 3 min, and the supernatant was filtered through an aqueous-phase filter membrane before HPLC analysis. The HPLC conditions were as follows: chromatographic column: Shodex RSpak KC-811 column (Showa Denko K.K., Tokyo, Japan) (8 mm × 300 mm); mobile phase: 3 mol/L perchloric acid solution, filtered and degassed; column temperature: 50 °C; injection volume; 5 μL; detection wavelength: 210 nm; flow rate: 1 mL/min. Another portion of the filtrate was used to measure ammonia nitrogen content using the phenol-sodium hypochlorite colorimetric method [15].

### 2.3. Microbiological Analysis Methods

#### 2.3.1. Determination of Viable Microbial Counts

Twenty grams of fresh whole-plant maize (raw and ensiled) were added to 180 mL of sterile physiological saline and shaken in a shaking incubator (B7 Bo’aosi General Shaking Incubator, Shanghai, China) at 120 rpm for 2 h at 37 °C. The mixture was then allowed to stand. One milliliter of the supernatant was transferred to a test tube containing 9 mL of sterile physiological saline and thoroughly mixed. Serial dilutions were prepared using sterile physiological saline.

A 100 μL aliquot of the 10^−6^ and 10^−7^ dilutions was spread onto MRS agar, malt extract agar, nutrient agar (NA), and mold medium (all purchased from Qingdao Haibo Biotechnology Co., Ltd., Qingdao, China), and incubated in an inverted position at 37 °C for 48–72 h. Simultaneously, 100 μL of the same dilutions were spread onto reinforced Clostridial agar medium (Qingdao Haibo Biotechnology Co., Ltd., Qingdao, China), placed in anaerobic gas packs (Mitsubishi Gas Chemical Company, Inc., Tokyo, Japan) with CO_2_ gas generators (Mitsubishi Gas Chemical Company, Inc., Japan), and incubated in an inverted position in a GH4500 water-jacketed incubator (Tianjin Teste Instrument Co., Ltd., Tianjin, China) at 37 °C for 48–72 h. Each dilution was tested in triplicate [16].

Colony Counting: Colonies were counted manually. Only plates with clearly distinguishable colonies and counts between 30 and 300 were used for quantification. The number of specific microorganisms per gram of fresh matter (FM), expressed as colony-forming units (CFU), was calculated using the following formula: Microbial count (CFU/g FM) = (Number of colonies × Dilution factor × 1000 μL)/Volume of diluted sample plated (μL).

#### 2.3.2. Determination of Microbial Community Diversity

A 0.5 g sample was weighed and ground in liquid nitrogen. Bacterial DNA was extracted using a bacterial genomic DNA extraction kit (DP302, Tiangen Biotech Co., Ltd., Beijing, China). DNA concentration and purity were assessed using a micro nucleic acid quantifier (HM-CWF1, Shandong Hengmei Electronic Technology Co., Ltd., Weifang, China). The nucleic acid concentration was required to exceed 10 ng/μL, with an optimal 260/280 absorbance ratio between 1.8 and 2.0. Qualified DNA samples were used for PCR amplification with universal bacterial primers 27F (5′-AGAGTTTGATCCTGGCTCAG-3′) and 1492R (5′-ACGGTTACCTTGTTACGACTT-3′). The PCR reaction mixture contained: 12.5 μL of 2× Taq Platinum PCR MasterMix, 1 μL of 10 μM forward primer (F), 1 μL of 10 μM reverse primer (R), 10 μL of DNA template (approximately 50–408 ng), and 1.5 μL of ddH_2_O. The PCR program was as follows: initial denaturation at 94 °C for 2 min; 30 cycles of denaturation at 94 °C for 30 s, annealing at 55 °C for 30 s, and extension at 72 °C for 1.5 min; followed by a final extension at 72 °C for 2 min. The remaining amplification product was stored at −80 °C. PCR products that passed electrophoresis were sent to Sangon Biotech Co., Ltd. (Shanghai, China) for sequencing. [7].

## 3. Results

### 3.1. Effects of Harvest Stage on Nutritional Quality and Clostridia of Whole-Plant Maize

#### 3.1.1. Effects of Harvest Stage on Nutritional Quality of Whole-Plant Maize

As shown in Table 2, the WSC content at the 2/3 ML stage was significantly lower than at the 1/3 ML stage (30.12 g/kg DM vs. 40.56 g/kg DM, *p* < 0.01), and significantly lower still at the ML stage compared to the 2/3 ML stage (15.58 g/kg DM vs. 30.12 g/kg DM, *p* < 0.01).

Starch content significantly increased during the maturation of whole-plant maize, rising by 34.0% from the 1/3 ML to the 2/3 ML stage (27.96 g/kg DM vs. 20.87 g/kg DM, *p* < 0.01), and by 13.4% from the 2/3 ML to the ML stage (31.70 g/kg DM vs. 27.96 g/kg DM, *p* < 0.01). No significant differences were observed in DM, NDF, ADF, or crude protein content (*p* > 0.05).

#### 3.1.2. Impact of Harvest Stage on the Number of *Clostridium* in Whole-Plant Corn

As shown in Table 3, there were no significant differences (*p* > 0.05) in the counts of lactic acid bacteria, aerobic bacteria, yeasts, *Clostridium*, or molds among the different harvest stages.

#### 3.1.3. Impact of Harvest Stage on the Relative Abundance of *Clostridium* in Whole-Plant Corn

**Figure 1:**
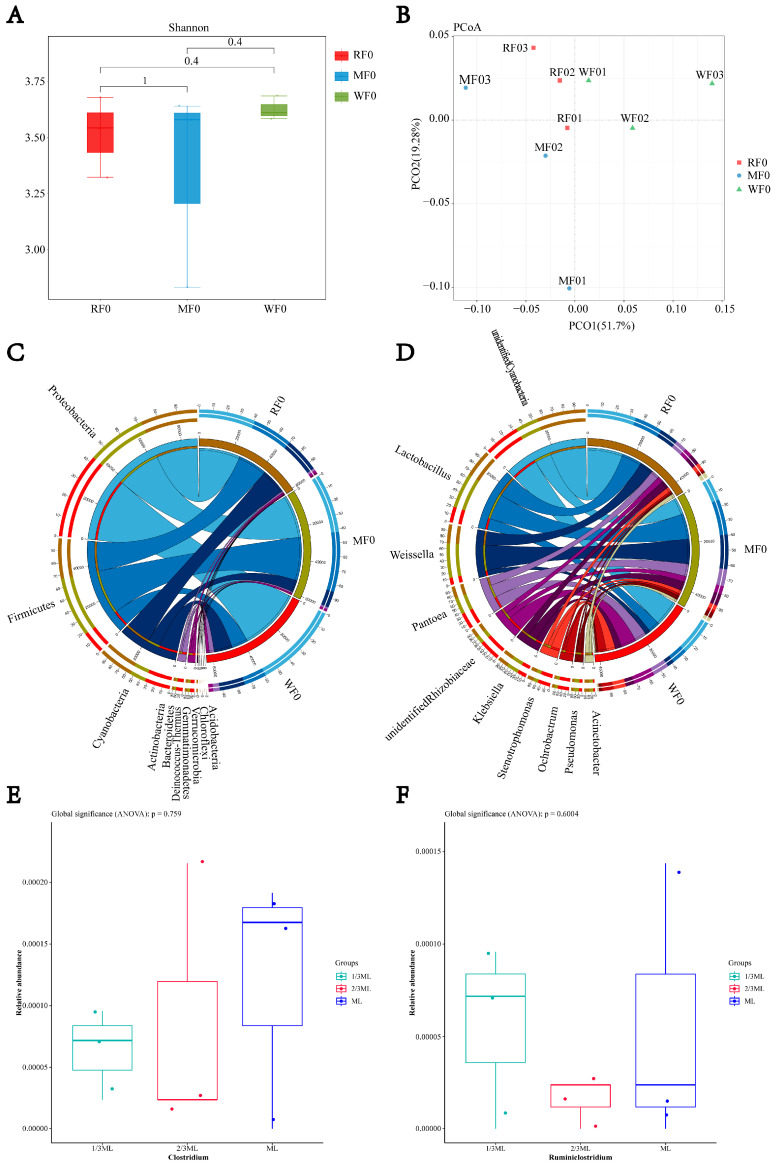
The effect of harvest period on the relative abundance of *Clostridium* in whole-plant Maize. (**A**) Alpha diversity; (**B**) PCoA analysis; (**C**) relative abundance of bacteria at the phylum level; (**D**) relative abundance of bacteria at the genus level; (**E**) relative abundance of *Clostridium*; (**F**) relative abundance of *Ruminiclostridium*. RF0: 1/3 ML stage; MF0: 2/3 ML stage; WF0: maturity stage.

As shown in Figure 1, at the phylum level, *Proteobacteria* (relative abundance 40.83–58.98%), *Firmicutes* (15.87–42.93%), and *Cyanobacteria* (11.84–25.20%) were predominant. At the genus level, the main genera included *Lactobacillus* (7.88–18.87%), *Weissella* (3.5–17.69%), and *Pantoea* (5.93–8.42%). *Clostridium* was detected across all harvest stages (1/3 ML, 2/3 ML, and ML) with low relative abundance (0.006–0.012%, *p* > 0.05). The relative abundance of *Ruminiclostridium* at 2/3 ML was lower than at 1/3 ML (0.002% vs. 0.006%, *p* > 0.05).

### 3.2. Impact of Compaction Density on Whole-Plant Corn Silage Quality and Clostridium

#### 3.2.1. Impact of Compaction Density on the Nutritional Composition of Whole-Plant Corn Silage

As shown in Table 4, the DM content in the high-density group (700 kg/m^3^) was significantly higher than that in the low-density group (350 kg/m^3^) on days 1, 7, 15, 30, and 60 of fermentation, with differences of 0.32%, 3.63%, 7.71%, 7.82%, and 6.61%, respectively (*p* < 0.05). After 60 days of ensiling, DM loss was significantly lower in the high-density group compared to the low-density group (3.37% vs. 9.39%, *p* < 0.05). Compared to day 1, WSC content in the high-density group significantly decreased after 60 days of fermentation (7.31 g/kg vs. 18.88 g/kg, *p* < 0.05). Similarly, the WSC content in the low-density group also showed a significant decrease (5.95 g/kg vs. 18.25 g/kg, *p* < 0.05).

#### 3.2.2. Impact of Compaction Density on the Fermentation Quality of Whole-Plant Corn Silage

As shown in Table 5, from day 7 to day 60 of fermentation, the lactic acid content in the high-density group was significantly higher than in the low-density group by 14.29%, 10.00%, 8.33%, and 9.68%, respectively (*p* < 0.01). On days 7 and 15, the ammonia-N content in the high-density group was significantly lower than that in the low-density group by 20.00% and 16.67%, respectively (*p* < 0.01). On days 30 and 60, the butyric acid content in the high-density group decreased by 56.25% and 50%, respectively, compared to the low-density group (*p* < 0.01). The pH of the high-density group on day 60 was significantly lower than on day 1 (3.80 vs. 5.30, *p* < 0.05).

Similarly, the low-density group showed a significant pH decrease on day 60 compared to day 1 (3.90 vs. 5.50, *p* < 0.05). Compared to day 1, the high-density group exhibited significant increases on day 60 in lactic acid (6.80 vs. 1.03 g/kg, *p* < 0.05), acetic acid (1.33 vs. 0.31 g/kg, *p* < 0.05), butyric acid (0.09 vs. 0.01 g/kg, *p* < 0.05), and lactic acid/acetic acid ratio (5.11 vs. 3.32, *p* < 0.05).

#### 3.2.3. Impact of Compaction Density on the Number of *Clostridium* in Whole-Plant Corn Silage

As shown in Table 6, on days 30 and 60 of fermentation, the number of *Clostridium* in the high-density group was significantly lower than in the low-density group, decreasing by 24.17% and 16.35%, respectively (*p* < 0.01). Additionally, on days 7, 15, 30, and 60, the number of lactic acid bacteria in the high-density group was significantly higher than in the low-density group by 14.48%, 6.95%, 6.39%, and 6.34%, respectively (*p* < 0.05).

#### 3.2.4. Impact of Compaction Density on the Relative Abundance of *Clostridium* in Whole-Plant Corn Silage

**Figure 2:**
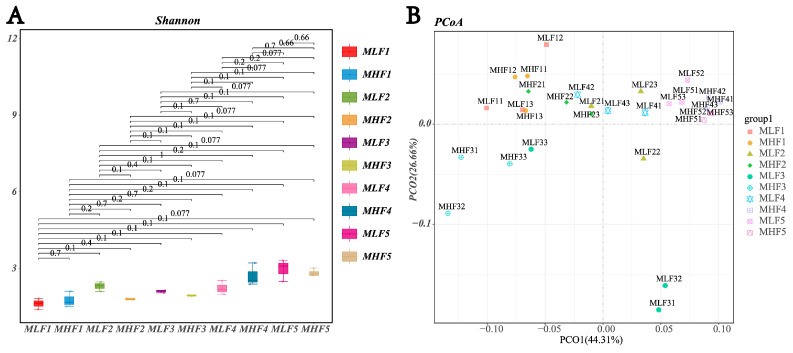

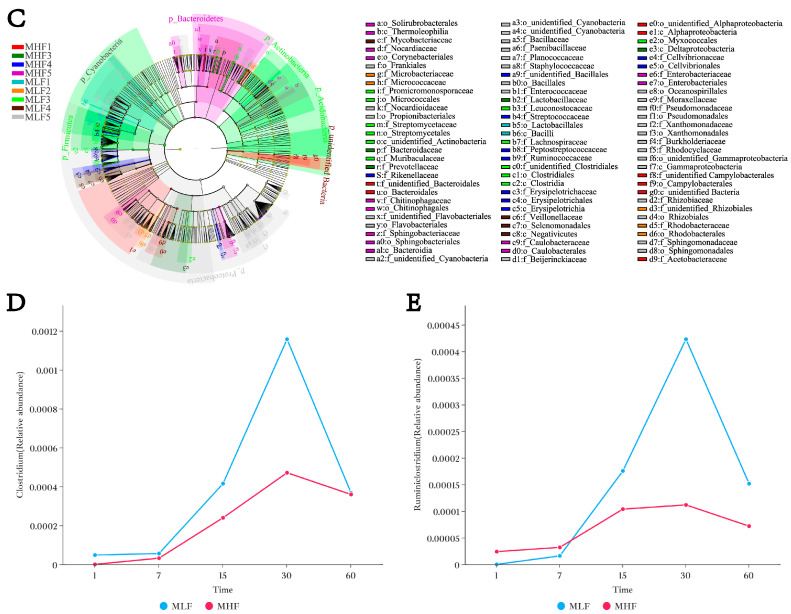
The effect of compaction density on the relative abundance of spoilage *Clostridium* in whole-plant maize. (**A**) Alpha diversity; (**B**) PCoA analysis; (**C**) Lefse multi-level taxonomic hierarchy. (**D**) Changes in the relative abundance of the genus *Clostridium* in MLF and MHF groups over the fermentation period. (**E**) Changes in the relative abundance of the genus *Ruminiclostridium* in MLF and MHF groups over the fermentation period. MLF: Whole-plant maize harvested at the 2/3 milk line stage and ensiled (silage fermentation). MFL1: fermentation 1 d (350 kg/m^3^); MFL1: fermentation 1 d (350 kg/m^3^); MFL2: fermentation 7 d (350 kg/m^3^); MFL3: fermentation 15 d (350 kg/m^3^); MFL4: fermentation 30 d (350 kg/m^3^); MFL5: fermentation 60 d (350 kg/m^3^). MHL1: fermentation 1 d (700 kg/m^3^); MHL2: fermentation 7 d (700 kg/m^3^); MHL3: fermentation 15 d (700 kg/m^3^); MHL4: fermentation 30 d (700 kg/m^3^); MHL5: fermentation 60 d (700 kg/m^3^).

As shown in Figure 2, there was no significant difference in the Shannon index among all groups (*p* > 0.05). However, microbial composition differed significantly among groups during days 1 to 60 of fermentation (*p* < 0.05). At the genus level, differential species in the MLF4 group included *Ruminiclostridium* (relative abundance 0.40%), *Anaerosinus* (0.40%), and *RummeliiBacillus* (1.17%). Notably, the relative abundance of *Ruminiclostridium* in the MLF4 group was significantly lower than in the MHF4 group (0.007% vs. 0.40%), representing a 98.25% decrease (*p* < 0.01). From days 1 to 30 of fermentation, the relative abundance of *Clostridium* gradually increased in both high- and low-density groups, peaking on day 30 (0.05% vs. 0.12%, *p* < 0.05), before decreasing as fermentation progressed. Furthermore, on day 30, the relative abundance of *Ruminiclostridium* in the high-density group was significantly lower than in the low-density group (0.12% vs. 0.40%, *p* < 0.05).

#### 3.2.5. Analysis of the Correlation Between Whole-Plant Corn Silage Fermentation Quality and *Clostridium*

**Figure 3:**
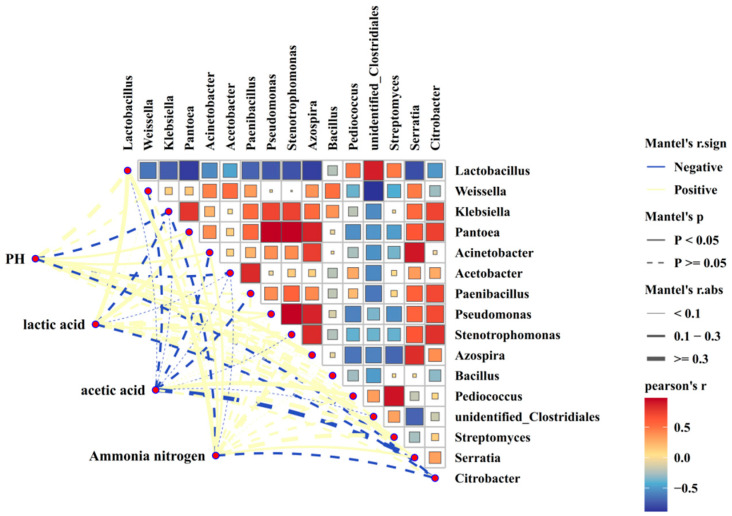
Correlation analysis between fermentation quality and spoilage Clostridium in whole-plant maize silage. in the correlation diagram, the color represents the correlation coefficient, r, which ranges from −0.5 to 0.5. r < 0 indicates a negative correlation, while r > 0 indicates a positive correlation.

As shown in Figure 2 and Figure 3, there was a negative correlation between putrefactive Clostridium and lactic acid bacteria (R^2^ = −0.58, *p* < 0.01). Putrefactive Clostridium was negatively correlated with pH, lactic acid, and acetic acid (R^2^ = −0.25, R^2^ = −0.23, R^2^ = −0.09, *p* > 0.05), and positively correlated with ammonia-N (R^2^ = 0.28, *p* > 0.05).

## 4. Discussion

The continuous increase in DM content during corn maturation results from the accumulation of DM in leaves, stems, and ears as the plant shifts from vegetative to reproductive stages [17]. Previous studies show that WSC are consumed for grain development and other metabolic activities during maturation. Consistent with this, our results reveal significantly lower WSC content at 2/3 ML and ML compared to 1/3 ML. ADF, linked to cellulose and lignin in cell walls [18], increases with plant senescence and lignification [17]. Accordingly, NDF and ADF gradually increased from 1/3 ML to ML, reflecting the conversion of green tissue to lignified material [19]. CP content decreases during maturation, as leaf protein synthesis is higher early on [20], but nitrogen is later redirected from stems and leaves to grains, lowering CP in vegetative parts [21]. Meanwhile, starch content increased steadily, paralleling grain development, since starch serves as the main storage compound during grain filling [22,23].

This study demonstrated that the high-density group effectively reduced DM loss in whole-plant corn silage by optimizing the anaerobic environment and promoting lactic acid fermentation throughout the fermentation process. Previous research indicates that high-density packing, by regulating the quantity and types of inoculated microorganisms, supports the dominance of beneficial lactic acid bacteria, thereby enhancing the anaerobic conditions and significantly minimizing DM loss during silage production [24]. This approach not only improves feed nutritional quality but also offers a more efficient preservation method for animal husbandry [25].

The fermentation quality of whole-plant corn silage is influenced by multiple factors, including compaction density, fermentation duration [26], and dynamic changes in microbial communities [27]. During ensiling, the metabolic activity and community structure of lactic acid bacteria play a direct role in determining fermentation quality [28]. Our results showed that lactic acid content in the high-density group was significantly higher than in the low-density group from day 7 to day 60 of fermentation, indicating that a high-density environment promotes substantial lactic acid accumulation. This occurs because the high-density packing rapidly establishes anaerobic conditions, allowing lactic acid bacteria to multiply swiftly under low-oxygen conditions [29], with lactic acid as the primary metabolic product of anaerobic fermentation [30]. Additionally, prolonged fermentation enhances the sustained activity of lactic acid bacteria, leading to continuous increases in lactic acid content while reducing acetic acid and ammonia-N production. Previous studies have found that with extended fermentation, lactic acid bacteria preferentially produce lactic acid via homofermentation rather than acetic acid via heterofermentation [31], which aligns with our experimental findings.

Compaction density is a critical factor in whole-plant corn silage production, significantly affecting microbial community structure and fermentation quality [32]. This study found that the number of *Clostridium* in the high-density group was significantly lower than in the low-density group on days 30 and 60 of fermentation, decreasing by 24.17% and 16.35%, respectively. This inhibition likely results from the rapid growth and acid production by lactic acid bacteria under high-density conditions, which cause a swift pH drop, creating an acidic environment unfavorable for *Clostridium* growth. However, micro-environmental variations, such as localized aerobic and strictly anaerobic zones within high-density silage, may cause uneven fermentation and prevent complete suppression of spoilage bacteria in certain areas [33]. The relative abundance of *Ruminiclostridium* decreased by 50% in the high-density group, possibly due to the combined inhibitory effects of fluctuating local redox conditions and the acidic environment. Previous research indicates that the growth and metabolism of this anaerobic microorganism are highly sensitive to environmental redox potential [34]. When local redox status shifts, especially toward a more oxidative state, the activity and proliferation of anaerobes like *Ruminiclostridium* are inhibited [35]. Additionally, an acidic environment (lower pH) stresses this genus by compromising cell membrane stability and metabolic enzyme function, leading to a decline in their population [36].

The results of this study revealed a negative correlation between *Clostridium* and lactic acid bacteria, suggesting that lactic acid bacteria may produce antimicrobial substances such as hydrogen peroxide and extracellular polypeptides that inhibit *Clostridium* [37]. The negative correlations between *Clostridium* and pH, lactic acid, and acetic acid indicate that the accumulation of these organic acids helps maintain a low pH, thereby suppressing the growth of *Clostridium*. Previous research has demonstrated that when silage pH drops below 4.5, the growth of *Clostridium* is effectively controlled. This rapid acidification is primarily driven by lactic acid bacteria, whose lactic acid production enhances environmental acidity, creating unfavorable conditions for anaerobic spore-forming bacteria like *Clostridium* [38]. Additionally, the study identified a positive correlation between *Clostridium* and ammonia-N, likely because *Clostridium* decomposes proteins and amino acids, producing ammonia-N. This suggests that higher *Clostridium* populations lead to increased protein degradation, raising ammonia-N concentrations. Therefore, lowering pH and promoting lactic acid bacteria growth in corn silage provides a theoretical foundation for inhibiting *Clostridium* and improving silage quality.

## 5. Conclusions

Harvesting period and compaction density significantly impact the fermentation quality and microbial community structure of whole-plant corn silage. High compaction density helps reduce dry matter loss and significantly inhibits the proliferation of Clostridium. Within the first 30 days of fermentation, the relative abundance of Clostridium rapidly increases, reaching a peak on day 30, and then gradually decreases. On day 30, the Clostridium abundance in the high-density group was significantly lower than that in the low-density group, indicating a stronger inhibitory effect of high density. Concurrently, high compaction density promotes the growth of lactic acid bacteria, which helps optimize the fermentation environment and improve silage quality.

## Figures and Tables

**Table 1:** Loading amount of fermenters for each treatment.

Treatment	Fermenter Volume (m^3^)	Loading Density (kg/m^3^)	Filling Quantity (kg)
Low packing density (MLF)	25.00	350	8.75
High packing density (MHF)	25.00	700	17.5

**Table 2:** Nutrient composition analysis of whole-plant maize.

Items	1/3 ML (Mean ± SD)	2/3 ML (Mean ± SD)	ML (Mean ± SD)
DM, g/kg FW	27.39 ± 6.64	31.50 ± 2.06	33.21 ± 0.67
WSC, g/kg DM	40.56 ± 2.78 ^A^	30.12 ± 3.82 ^B^	15.58 ± 2.35 ^C^
NDF, g/kg DM	40.17 ± 1.03	42.77 ± 1.06	45.12 ± 2.14
ADF, g/kg DM	22.44 ± 2.10	23.95 ± 0.55	25.00 ± 0.71
Starch, g/kg DM	20.87 ± 0.83 ^C^	27.96 ± 0.42 ^B^	31.70 ± 0.62 ^A^
CP, g/kg DM	9.16 ± 0.44	8.78 ± 0.23	7.78 ± 0.34

Note: 1/3 ML: 1/3 milk line stage; 2/3 ML: 2/3 milk line stage; ML: mature stage. Different capital letters above the means indicate significant differences (*p* < 0.01). Crude protein: CP.

**Table 3:** The effect of harvest period on the microbial count of whole-plant maize.

Items	Period		
Microbial Count (log_10_ CFU/g FW)	1/3 ML (Mean ± SD)	2/3 ML (Mean ± SD)	ML (Mean ± SD)
Lactic acid bacteria	4.74 ± 0.22	4.64 ± 0.29	4.44 ± 0.48
Aerobic bacteria	6.51 ± 0.29	6.52 ± 0.28	6.26 ± 0.29
Yeasts	4.82 ± 0.19	4.83 ± 0.18	4.68 ± 0.15
Clostridium	2.62 ± 0.12	2.63 ± 0.11	2.67 ± 0.07
Molds	2.44 ± 0.28	2.45 ± 0.29	2.45 ± 0.30

Note: 1/3 ML: 1/3 milk line stage: 2/3 ML: 2/3 milk line stage: ML: mature stage.

**Table 4:** The effect of compaction density on the nutritional components of whole-plant corn silage.

Item	Treatment	Days of Ensiling					SEM	*p*-Value		
		1	7	15	30	60		Day	ρ	D × ρ
DM	350 kg/m^3^	31.27 ^B^	29.51 ^B^	28.29 ^B^	28.25 ^B^	28.33 ^B^	0.52	<0.326	<0.01	<0.01
	700 kg/m^3^	31.37 ^A^	30.58 ^A^	30.47 ^A^	30.46 ^A^	30.20 ^A^				
CP (g/kg DM)	350 kg/m^3^	7.34	7.34	7.26	7.29	7.20	0.03	<0.425	0.915	<0.01
	700 kg/m^3^	7.32	7.34	7.27	7.30	7.21				
NDF (g/kg DM)	350 kg/m^3^	42.72	42.26	41.57	42.13	41.13	0.28	<0.426	0.360	<0.01
	700 kg/m^3^	42.68	42.25	41.33	41.50	41.02				
ADF (g/kg DM)	350 kg/m^3^	22.87	22.90	21.78	19.46	17.95	0.98	<0.125	0.484	<0.01
	700 kg/m^3^	22.47	21.95	20.83	19.12	18.16				
WSC (g/kg DM)	350 kg/m^3^	18.88 ^a^	11.12 ^bc^	8.56 ^bd^	7.76 ^bd^	7.31 ^bd^	2.14	<0.01	0.408	<0.01
	700 kg/m^3^	18.25 ^a^	8.84 ^b^	7.10 ^c^	6.46 ^d^	5.95 ^e^				

Note: For each item, different uppercase letters as superscripts in the same column indicate a significant difference (*p* < 0.05); different lowercase letters as superscripts in the same row indicate a significant difference (*p* < 0.05). ρ: density; D × ρ: interaction between days and density. Crude protein: CP.

**Table 5:** The Effect of compaction density on the fermentation quality of whole-plant maize silage.

Items	Treatment	Days of Ensiling					SEM	*p*-Value		
		1	7	15	30	60		Day	ρ	D × ρ
pH	350 kg/m^3^	5.50 ^a^	4.20 ^b^	4.00 ^b^	3.90 ^b^	3.90 ^b^	0.26	<0.01	0.537	<0.01
	700 kg/m^3^	5.30 ^a^	4.00 ^b^	3.90 ^b^	3.80 ^b^	3.80 ^b^				
LA (%DM)	350 kg/m^3^	0.80 ^b^	3.50 ^Bb^	5.00 ^Bb^	6.00 ^Ba^	6.20 ^Ba^	0.09	<0.01	0.553	<0.01
	700 kg/m^3^	1.03 ^b^	4.00 ^Ab^	5.50 ^Ab^	6.50 ^Aa^	6.80 ^Aa^				
AC (% DM)	350 kg/m^3^	0.20 ^be^	0.50 ^bd^	0.80 ^bc^	1.20 ^b^	1.50 ^a^	0.59	<0.01	0.572	<0.01
	700 kg/m^3^	0.31 ^bd^	0.40 ^bd^	0.70 ^bc^	1.00 ^b^	1.33 ^a^				
NH3-N (%DM)	350 kg/m^3^	0.05 ^bc^	0.15 ^Ab^	0.18 ^Ab^	0.20 ^a^	0.22 ^a^	0.98	<0.01	0.296	<0.01
	700 kg/m^3^	0.03 ^c^	0.12 ^Bb^	0.15 ^Bb^	0.18 ^a^	0.20 ^a^				
BA (%DM)	350 kg/m^3^	0.03 ^d^	0.07 ^c^	0.12 ^b^	0.16 ^Aa^	0.18 ^Aa^	0.02	<0.01	<0.01	<0.01
	700 kg/m^3^	0.01	0.02	0.05	0.07 ^B^	0.09 ^B^				
LA/AC	350 kg/m^3^	4.00 ^c^	7.00 ^a^	6.25 ^a^	5.00 ^b^	4.13 ^c^	0.64	<0.01	0.073	<0.01
	700 kg/m^3^	3.32 ^e^	10.00 ^a^	7.86 ^b^	6.50 ^c^	5.11 ^d^				

Note: For each item, different uppercase letters as superscripts in the same column indicate a significant difference (*p* < 0.05); different lowercase letters as superscripts in the same row indicate a significant difference (*p* < 0.05). ρ: density; D × ρ: interaction between days and density. Lactic acid: Butyric acid: LC; Acetic acid: AC.

**Table 6:** The effect of compaction density on the nutritional components of whole-plant maize silage.

Items	Treatment	Days of Ensiling					SEM	*p*-Value		
		1	7	15	30	60		Day	ρ	D × ρ
Microbial count (log10 CFU/g FW)	350 kg/m^3^	4.80 ^aD^	6.62 ^bC^	7.19 ^bB^	7.83 ^bB^	8.20 ^bA^	1.23	<0.01	0.506	<0.01
	700 kg/m^3^	4.14 ^bC^	7.58 ^aB^	7.69 ^aB^	8.33 ^aA^	8.72 ^aA^				
Lactic acid bacteria	350 kg/m^3^	6.40 ^aA^	5.23 ^B^	5.17 ^B^	5.00 ^aB^	3.46 ^bC^	2.56	<0.01	0.995	<0.01
	700 kg/m^3^	6.07 ^bA^	5.74 ^B^	5.00 ^B^	4.47 ^bC^	3.97 ^aD^				
Aerobic bacteria	350 kg/m^3^	4.24	4.48	4.24	4.30	4.17	0.63	<0.01	0.079	<0.01
	700 kg/m^3^	4.50 ^A^	4.46 ^A^	4.10 ^A^	3.80 ^B^	3.77 ^B^				
Yeasts	350 kg/m^3^	2.63 ^B^	2.60 ^B^	2.60 ^B^	3.60 ^aA^	3.73 ^aA^	0.55	<0.01	0.048	<0.01
	700 kg/m^3^	2.43 ^B^	2.62 ^B^	2.70 ^B^	2.73 ^bB^	3.12 ^bA^				
Clostridium	350 kg/m^3^	2.48 ^B^	3.02 ^A^	2.57 ^B^	2.00 ^B^	1.53 ^C^	0.82	<0.01	0.357	<0.01
	700 kg/m^3^	2.43 ^B^	2.49 ^B^	2.57 ^A^	2.03 ^C^	1.17 ^D^				

Note: For each item, different uppercase letters as superscripts in the same column indicate a significant difference (*p* < 0.05); different lowercase letters as superscripts in the same row indicate a significant difference (*p* < 0.05). ρ: density; D × ρ: interaction between days and density.

## Data Availability

The original contributions presented in this study are included in the article. Further inquiries can be directed to the corresponding author.
